# A Novel Biallelic *STING1* Gene Variant Causing SAVI in Two Siblings

**DOI:** 10.3389/fimmu.2020.599564

**Published:** 2021-01-08

**Authors:** Malak Ali Alghamdi, Jaazeel Mulla, Narjes Saheb Sharif-Askari, Francisco J. Guzmán-Vega, Stefan T. Arold, Mervat Abd-Alwahed, Nasser Alharbi, Tarek Kashour, Rabih Halwani

**Affiliations:** ^1^ Department of Pediatrics, Medical Genetic Division, College of Medicine, King Saud University, Riyadh, Saudi Arabia; ^2^ Department of Pediatrics, College of Medicine, King Saud University, Riyadh, Saudi Arabia; ^3^ Sharjah Institute of Medical Research, College of Medicine, University of Sharjah, Sharjah, United Arab Emirates; ^4^ Computational Bioscience Research Center (CBRC), Division of Biological and Environmental Sciences and Engineering (BESE), King Abdullah University of Science and Technology (KAUST), Thuwal, Saudi Arabia; ^5^ College of Medicine Research Center, King Saud University, Riyadh, Saudi Arabia; ^6^ Department of Pediatrics, Pulmonology Division, College of Medicine, King Saud University, Riyadh, Saudi Arabia; ^7^ Cardiology Department, College of Medicine, King Saud University, Riyadh, Saudi Arabia; ^8^ Department of Clinical Sciences, Sharjah Institute for Medical Research (SIMR), College of Medicine, University of Sharjah, Sharjah, United Arab Emirates

**Keywords:** interferonopathies, pulmonary inflammation, SAVI, JAK inhibitor, STING-associated vasculopathy of infancy, cutaneous vasculopathy, STING1

## Abstract

STING-associated vasculopathy of infantile-onset (SAVI) is one of the newly identified types of interferonopathies. SAVI is caused by heterozygous gain-of-function mutations in the *STING1*. We herein report for the first time a homozygous variant in the *STING1 gene* in two siblings that resulted in constitutive activation of *STING* gene and the SAVI phenotype. Exome sequencing revealed a novel homozygous NM_198282.3: c.841C>T; p.(Arg281Trp) variant in exon 7 of the *STING1* gene. The variant segregated in the family to be homozygous in all affected and either heterozygous or wild type in all healthy. Computational structural analysis of the mutants revealed changes in the STING protein structure/function. Elevated serum beta-interferon levels were observed in the patients compared to the control family members. Treatment with Janus kinase inhibitor (JAK-I) Ruxolitinib suppressed the inflammatory process, decreased beta-interferon levels, and stopped the progression of the disease.

## Introduction

Stimulator of interferon genes (STING), encoded by the *STING1* gene, is a facilitator of innate immune signaling which acts as a sensor of cytosolic viral and bacterial DNA and promotes the production of type I interferon (IFN-α and IFN-β) (1). STING-associated vasculopathy of infantile onset (SAVI) is a rare autosomal dominant genetic disorder classified under the newly discovered type 1 interferonopathies. More than twenty case reports have described variable SAVI phenotypes, and eleven heterozygous gain-of-function variants were so far reported (2). Such diseases, despite being caused by different mutations, manifest similarly with widespread chronic inflammation affecting primarily the skin and lungs (2). Classically, all reported cases presented with cutaneous vasculopathy, pulmonary inflammation, increased immunoglobulins with inflammatory markers, interferon signature and negative cultures (2, 3). The presence of this pattern of signs and symptoms in association with failure to thrive (FTT) and recurrent fever should indicate a *STING1* gene study (4, 5). Traditional treatment with immunomodulators has failed repeatedly (2, 6, 7). A heterozygous STING-activating variant was described first in 2014 in a familial inflammatory syndrome, and since then, more than 20 case reports have described variable SAVI phenotypes and eleven heterozygous gain-of-function (GoF) variants (2, 6, 8–15). A Janus kinase inhibitor (JAK-I) was given to several SAVI patients and resulted in different clinical outcomes, as shown in **Supplementary Table 2** (3, 6, 10, 13, 15–18).

Here, we report two siblings born to a consanguineous couple of Syrian descent who presented with the classical picture of cutaneous vasculopathy and severe pulmonary hypertension (HTN) with significant parenchymal lung disease. Whole exome sequencing (WES) confirmed a novel homozygous mutation, in the *STING1* gene in both affected siblings resulting in constitutive activation of *STING1* gene and the development of SAVI phenotype.

## Methods

### Patients

Written informed consent was provided for all family members study participants; for minors’ participants, one or both parents provided written informed consent, and the parents provided written informed consent themselves.

### Exome Sequencing

Approximately 37 Mb (214,405 exons) of the consensus coding sequences (CCSs) were enriched from fragmented genomic DNA by >340,000 probes that were designed against the human genome (Nextera Rapid Capture Exome, Illumina), and the generated library was sequenced on an Illumina NextSeq or HiSeq 4000 platform (Illumina) to an average coverage depth of 70–100X. An end-to-end in-house bioinformatics pipeline, including base calling, primary filtering of low-quality reads and probable artifacts, and annotation of variants, was applied.

All of the disease-causing variants that had been reported in ClinVar (class 1) as well as all variants with a minor allele frequency (MAF) of less than 1% in the ExAc database were considered for this study. Our evaluation focused on exons with intron boundaries ±20.

All of the relevant inheritance patterns were considered, and the family history and clinical information that was provided were used to evaluate the variants that we eventually identified. Only the variants that were related to the phenotype are reported.

### Segregation Analysis by PCR

The coding sequence of *STING1* exon 7 was amplified by using designed primers on the genomic DNA to validate the variant by Sanger sequencing in both the forward and reverse directions to exclude NGS artifacts. The parents and all of the siblings (see **Figure 3**) were tested for the presence of the variant to complete the family segregation and confirm the pathogenicity.

### Sanger Sequencing

The reference sequence used for numbering was *STING1* (NM_198282.3) and the sequencing primer was designed using the genomic sequence (NG_034249) to amplify exon 7 and the flanking two introns. Genomic DNA was extracted from the whole family blood samples using QIAamp blood extraction Kit (Qiagen, Dusseldorf, Germany) and was amplified in first PCR amplification reaction using Hotstar Taq plus Master Mix (Qiagen). PCR products underwent a second PCR reaction with BigDye Terminator v3.1 Cycle Sequencing Kit (Applied Biosystems, Foster City, CA). The products of sequencing reaction were purified by ethanol/EDTA precipitation and directly sequenced in both directions to get consensus sequences using ABI 3130 Genetic Analyzer (Applied Biosystems).

### Computational Structural Analysis of the Mutants

The crystal structure of the human STING C-terminal domain bound to cyclic di-GMP (PDB accession number 4EMT) was used as a basis for manual evaluation of the NM_198282.3: p.(Arg281Trp) variant using the Pymol program (pymol.org).

### qRT-PCR Relative Quantification for Gene Expression

Total RNA was extracted from PBMCs using RNeasy mini kit (Qiagen) following manufacturer’s instructions. Equivalent amounts of total RNA (10 ng) were reverse-transcribed and target genes were amplified in one-step reaction using Quantitect SYBR Green kit (Qiagen). Target genes were normalized to the GAPDH gene and relative concentrations were determined using ΔΔct method in software of rotor-gene Q5 plex (Qiagen). Primer sequences used are provided in the **Supplementary Table 1**.

### Ruxolitinib Treatment

MA was treated with ruxolitinib, a JAK-I. He was given a dose of 5 mg BID over a span of 6 months. Immunological and blood assessments were performed pre- and posttreatment, summarized in **Table 1**, to compare the effect of ruxolitinib on SAVI. With the promising clinical improvement observed, YA was soon started with a dose of 5 mg BID.

**Table 1:** Immunological markers and baseline blood work before and after treatment with a JAK-1 Inhibitor for MA and YA.

		Case 1: MA		Case 2: YA	
		Pretreatment	Posttreatment	Pretreatment	Posttreatment
Immunological Markers	IgA	2.050	1.720	2.610	NA
	IgG	18.70	16.40	16.70	NA
	IgM	0.923	0.699	0.873	NA
	Interferon β (pg/ml)	0.23 (81.8)	0.012 (70)	10.9	NA
Blood Work	CRP	14.7	5.9	7.60	3.190
	ESR	60	30	54	44
	BNP	3090	78	NA	NA
	CK	32	NA	NA	NA
	Trop1	12.4	6.3	NA	NA

### IFN- β1 Assessment Using ELISA Pre- and Posttreatment

A quantitative sandwich ELISA for IFN-β1 (SAB) was performed according to the manufacturer’s instructions. Briefly, a standard curve for human IFN-β1 was prepared (range 0-1000 pg/ml). One hundred microliters of each standard, sample (plasma) and blank control were added to wells precoated with a biotin-conjugated polyclonal antibody specific to IFN-β1. After incubation for 2 h at 37°C, the liquid in the wells was removed. Without wash, 100 µl of Detection Reagent A was added and incubated for 1 h at 37°C. After three washes, 100 µl of Detection Reagent B was added to the wells and incubated for 1 h at 37°C. Following five washes, 90 µl of chromogen TMB substrate solution was added and incubated for 30 min at 37°C and protected from light. Only those wells that contained IFN-β1, biotin-conjugated antibody and enzyme-conjugated avidin exhibited changes in color. The enzyme-substrate reaction was stopped using 90 µl of sulfuric acid solution, and the absorbance of each well was read at 450 nm using a Synergy 2 microplate reader (BioTek, Winooski, Vermont, USA).

## Results

**Figure 1:**
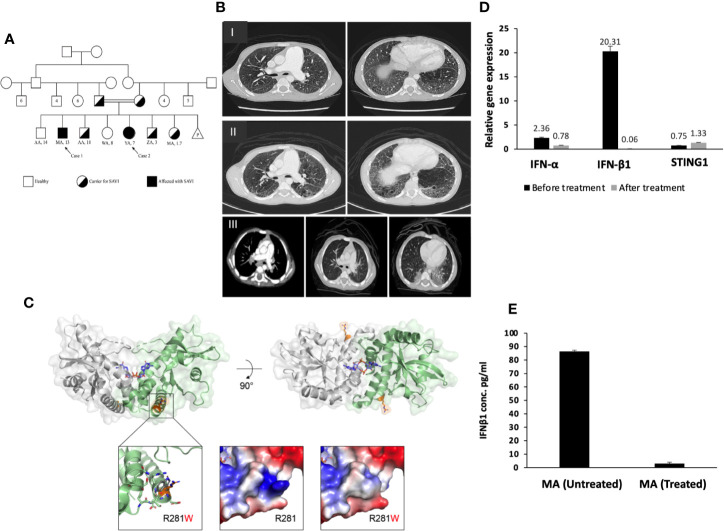
**(A)** Pedigree of the SAVI patients’ family. **(B–I)** Chest CT for MA, showing diffused centrilobular emphysema and interlobar septal thickening in both lung fields associated with enlarged main pulmonary artery and its main branches. **(B–II)** Interval progression of diffuse emphysematous changes more marked in both lower lobes with honeycombing. Changes of pulmonary arterial hypertension. **(B–III)** CT chest of YA, showing diffused fine nodular opacities bilaterally with consolidation in both lungs bases posteriorly. The pulmonary trunk and major mediastinal vessels are unremarkable. **(C)** Crystallized structure of the human STING C-terminal domain bound to cyclic-di-GMP (PDB accession 4EMT). Top left: Front view of the STING dimer, with the view axis parallel to the membrane. The cyclic-di-GMP is shown as blue sticks in the center of the dimerization interface. R281 is shown as orange sticks. Top right: View of the STING dimer from the top, showing both appearances of R281. Bottom left: Zoom into the proximity of R281, showing neighboring residues that contribute to the presence of the hydrophilic patch on the protein surface and that might present clashes with the tryptophan substitution (shown as gray sticks). Bottom center: Surface electrostatic potential surrounding R281, with the protruding positive patch generated by the arginine, which is lost in the bottom-right picture after the substitution for tryptophan. **(D)** Gene expression for STING1, IFN-α and IFN-β1 before and after treatment for patient MA. There is overexpression for IFN-β1 in patients compared to non-affected individuals (SEM = 3.4). STING1 and IFN-α showed no significant increase in expression in patients compared to non-affected individuals. STING1 (SEM = 0.44) and IFN-α (SEM = 3.4). **(E)** IFN-β1 plasma level of the patient MA before and after ruxolitinib treatment (SEM = 0.109).

**Figure 2:**
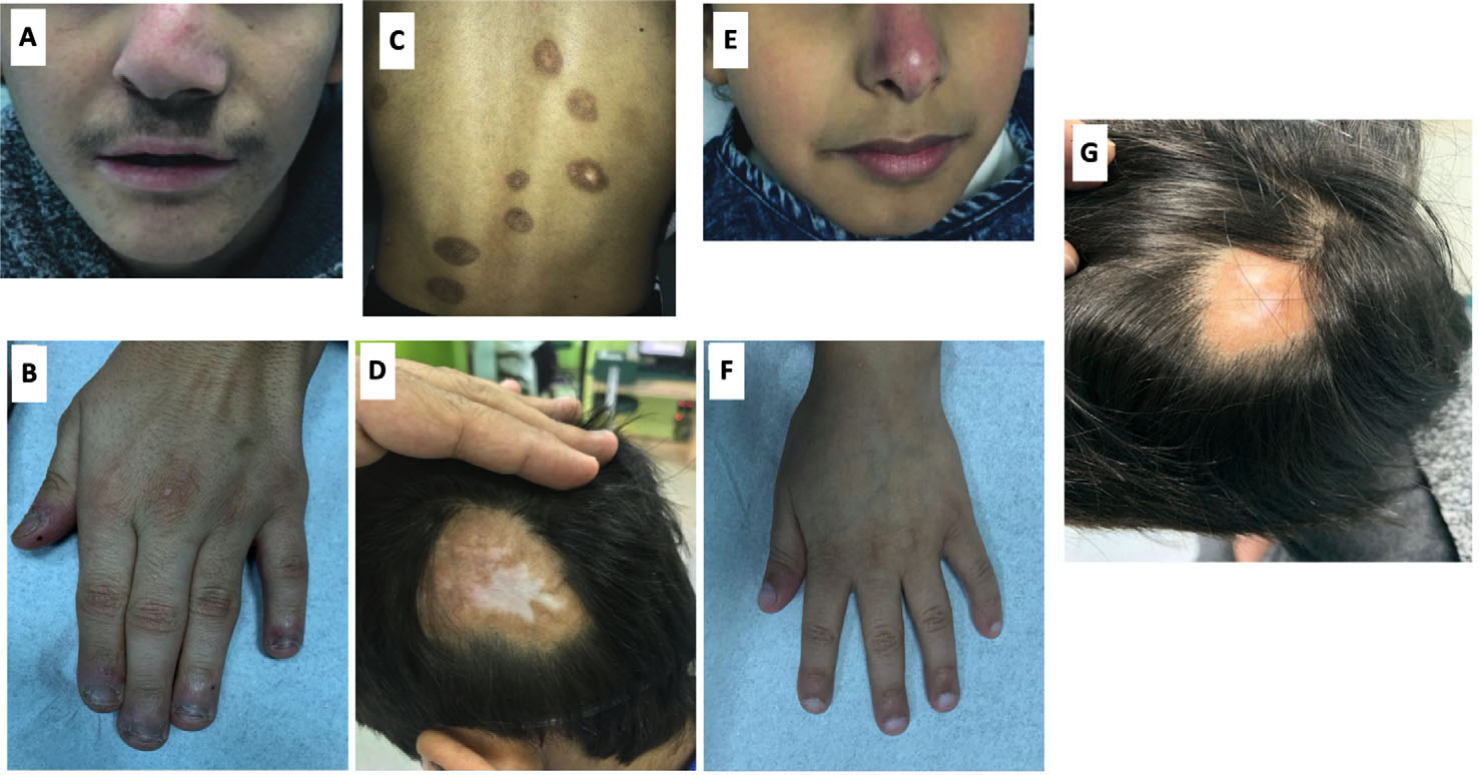
**(A)** Shows the gross phenotype of MA at 15 years of age and sister YA at 7 years of age. Figure shows the index case with nasal deviation. **(B)** Hand of MA showing mild acrocyanosis with leukonychia but no acro-necrosis. **(C)** shows eight healed hyperpigmented macules of various sizes on the back of MA, with a few showing central clearing. **(D)** Image of posterior-lateral scalp showing a large area of alopecia with central hypopigmentation. **(E)** YA with violaceous discoloration of the nasal cartilage. Pre-treatment; a large area of hair loss on the posterior lateral scalp with central hypopigmentation. **(F)** Hand of YA showing no clubbing or acro-necrosis. **(G)** Post-treatment; Reduction in the size of the area of hair loss with hair regrowth and a decrease in the hypopigmentation.

MA, a 13-year-old boy, and YA, a 7 years old girl, were diagnosed with congenital idiopathic pulmonary hypertension (HTN) at early age. No medical reports from their earlier years in Syria could be obtained. The family moved to Riyadh when MA was eight and YA was one-year-old. MA was repeatedly admitted to multiple hospitals in Riyadh due to frequent chest infections. At the age of 11, MA was admitted to King Saud University Medical City (KSUMC-KKUH) for the investigation of a violet-red skin rash (see **Figure 2**). All immunological workups showed no abnormalities. Two skin biopsies taken from the dermal lesions displayed a nonspecific inflammatory nature. A CT scan of the chest was performed and displayed centrilobular emphysema and pulmonary hypertension (**Figure 1B**). He also started to exhibit clubbing of the fingers and Raynaud’s phenomenon from cold exposure. Various trials of systemic steroids, antifungals and antibiotics failed to achieve any improvement in his condition. A year later, MA entered a state of dyspnea at rest and complete oxygen dependency. Repeated echocardiogram (ECHO) showed severe dilatation of the right ventricle with moderate right ventricular dysfunction, an ejection fraction (EF) of 31% with signs of severe pulmonary HTN. During multiple hospital visits, MA erythrocyte sedimentation rate (ESR) was high (a mean of 46 mm/hr; reference range: 0–15 mm/h), and C-reactive protein (CRP) was normal to high. The antinuclear antibody (ANA) titer was 1:160 with a negative anti-neutrophil cytoplasmic antibody (ANCA) titer.

Conversely, in her toddler years, YA developed arthritis mainly in her knees, ankles, elbows, and fingers. An associated malar rash was also noticed on her face with sunlight exposure along with Raynaud’s phenomenon with exposure to cold. No oral ulcers or other mucocutaneous lesions were found (**Figure 2E**). She had elevated antinuclear antibody (ANA) and double-stranded DNA (dsDNA) levels. The ANCA titer was elevated (1:640) with a perinuclear pattern (pANCA). Antibodies against proteinase 3 and myeloperoxidase were negative. A lung biopsy was performed and the findings were consistent with pulmonary HTN.

Fulfilling the criteria for systemic lupus erythematosus (SLE), she was started on a standard treatment protocol. A similar manifestation was previously reported for a patient with SAVI (8). Steroids, methotrexate, and hydroxychloroquine were prescribed in the outpatient settings at KSUMC-KKUH under the care of pediatric pulmonology and rheumatology teams. Her parents reported a mild improvement in her overall symptoms when receiving treatment for SLE. Despite her cough being described as more aggressive than her brother’s, she did not manifest any of the classical cutaneous vasculopathy lesions, exhibited less frequent chest infections, and did not require the use of home oxygen.

**Figure 3:**
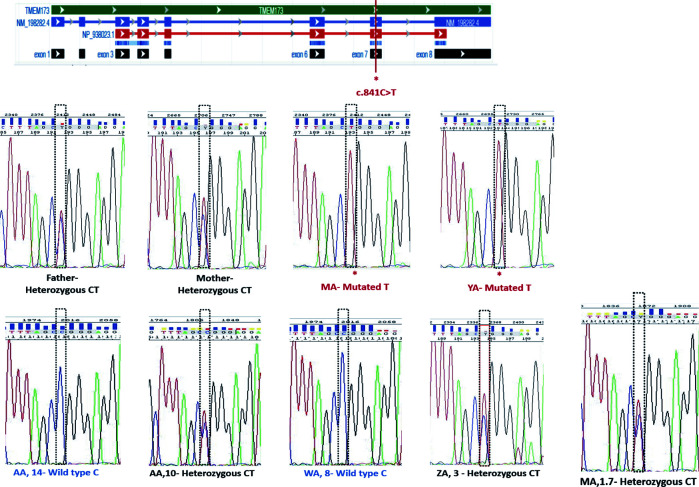
Segregation analysis for the STING1, NM_198282.3: c.841C>T variant.

WES analysis was conducted on both patients, parents, and siblings. Both MA and YA were found to have a homozygous variant, NM_198282.3: c.841C>T; p.(Arg281Trp), p.R281W of uncertain significance (class 3) on exon 7 in the *STING1* gene. This variant was predicted to be “deleterious” by SIFT, “probably damaging” by PolyPhen, and “disease causing” by Mutation Taster, suggestive of SAVI with a novel autosomal recessive mutation. Sanger sequencing was performed on the whole family members (father, mother, YA, MA, AA-het, ZA, WA, AA-hom.). Both parents and three of the siblings were heterozygous for the mutation (**Figure 1A**; **Figure 3**).

The crystallized structure of the human STING1 C-terminal domain (CTD) was analyzed to further characterize the mutation and its 3D environment. The mutated Arg281 is located on the surface of the STING1 cytosolic CTD (**Figure 1C**, top), capping an exterior helix and creating a patch with a positive charge protruding from the protein surface (**Figure 1C**, bottom) (19). The substitution for tryptophan does not introduce steric clashes and is tolerated from a structural point of view. Nonetheless, the substitution could mildly affect the protein structure/function by altering the conformation of its containing helix or the interaction with its neighboring helix, which is a part of the dimerization interface of STING and is also adjacent to the cyclic-di-GMP binding site (**Figure 1C**, top) (20). The substitution could also disturb the potential interaction of this region with one or more STING binding partners by changing the local charge on the protein surface (**Figure 1C**, bottom).

**Figure 4:**
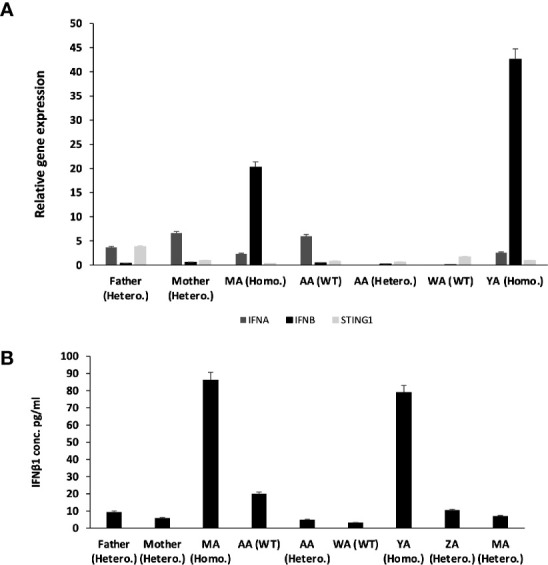
Relative Gene expression for STING1, IFNα and IFNβ1 for the whole family before treatment. **(A)** qRT-PCR relative Quantification for Gene Expression for STING1, IFNα and IFNβ1. There is over expression for IFNβ1 in patients compared to non-affected individuals (SEM = 3.4). STING1 and IFNα showed no significant increase in expression in patients compared to non-affected individuals. STING1 (SEM = 0.44) and IFNα (SEM = 3.4). **(B)** IFN β1 plasma level of the family before treatment.

The effect of this mutation on the expression and function of STING was then determined. No difference in *STING1* expression was observed in the patients compared to carrier or healthy control family members. However, the gene expression and plasma levels of IFN-β1 were significantly higher in the patients compared to carrier or non-carrier family controls (**Figure 4**), suggesting the constitutive activation of the STING pathway.

Prior to commencing therapy, brain MRI showed no abnormalities, and baseline blood work was within range (**Table 1**). Treatment with ruxolitinib, a JAK-I (21), was commenced for both MA and YA patients. MA was given a dose of 5 mg twice daily over a span of 6 months. After receiving the first dose, his parents reported a significant improvement in his lung and dermatological symptoms (see **Figures 2, G, H**). He no longer experienced dyspnea or the need for oxygen at rest. **Table 1** shows the immunological workup before and after the initiation of ruxolitinib, with a clear decline in immunoglobulin levels and acute phase reactants. Interestingly, treatment with Ruxolitinib suppressed the expression of IFN-β1 in patients’ PBMCs (**Figure 1D**) and dramatically lowered its serum levels (**Figure 1E**).

Today, MA and YA have clinically improved since the start of the JAK-1/2 inhibitor. They are performing daily activities while off oxygen. Their cognitive functions have never been affected, and they are able to perform many age-related tasks.

The CT scans of our patients’ chests showed typical findings of SAVI; however, the sequence of their symptoms does not align with the natural course of the disease, as described in the majority of cases reported. The patients reported here seemed to have acquired lung involvement far before the other clinical features of SAVI. We assume that this atypical presentation of a relatively rare genetic disease is the reason behind the delayed genetic testing and diagnosis. Compared to other reported cases, the two siblings reported here with the novel homozygous mutation had a milder form of the diseases.

## Discussion

In this study we report a novel homozygous variant causing the SAVI phenotype. Functional validation confirmed the gain-of-function nature of this biallelic *STING1 gene* variant with high serum immunoglobulins, IFN-β1 and inflammatory markers. Other heterozygous family members, however, were healthy and had no elevation of inflammatory cytokines (**Figure 4**). This suggests that, since STING present as a dimer, WT/Mut dimers may not cause GOF; while Mut/Mut levels in heterozygous patients may not be enough to cause inflammation. We demonstrated that treatment with JAK-I successfully corrected the clinical phenotype and immunological features.

A study published by Munoz et al. (2) in 2015 a patient with SAVI whose clinical picture mimicked that of childhood protracted superficial granulomatosis with polyangiitis (GPA) described the inflammatory process as involving the skin and beginning well before the age of six months. The pulmonary findings started later on during the disease course (2). Patients initially present with cutaneous vasculopathy in the form of red-violet plaques and lesions on the face and acral areas. These lesions worsen during winter or with cold exposure, especially at sites of lesser blood flow, often resulting in painful crust and eschar formation. Over time, with ongoing disease progression, dystrophy of the nails, nasal septal perforation, and gangrene of the digits from repeated infractions can be observed (2, 6). The same case report identified that such cutaneous lesion progression was the second of the three consecutive cutaneous phases of SAVI (2).

Surprisingly, of both of our patients, the older brother was the only one to manifest any skin lesions later on during the course of his disease. However, by that time, he had already suffered from ILD for nearly a decade. He had only a single flare of skin lesions that gradually healed over time; there were no other follow-up skin lesions or flares in winter or with exposure to lower temperatures. A similar presentation was reported in a patient with SAVI who presented initially with ILD (6). His sister, YA, on the other hand, exhibited only dermal involvement in the form of a malar rash on the face after sun exposure. That being said, such an isolated cutaneous lesion in the context of pulmonary involvement with a clinical history of failure to thrive and recurrent febrile attacks was reported in a single patient in 2014 found to have SAVI with lupus-like manifestations (8).

Recently, Lin et al. (22) reported same mutation (p.R281W) in six patients from four unrelated families. They showed that transfection of HEK293T cells with different STING1 constructs including the homozygous R281W and heterozygous R281Q variants led to increase in IFNB1 reporter activity. However, compared to heterozygous variant R281Q, they reported a lower autoactivation of STING with the homozygous R281W construct (22). In a case series of six patients reported as having SAVI in 2014 by Liu et al. (11) all six cases with pulmonary involvement had paratracheal lymphadenopathy, and five out of six had abnormal pulmonary function tests (PFTs) and computerized tomography (CT) evidence of ILD. The cause of death in patients reported to have SAVI was predominantly due to pulmonology complications in early adulthood (2, 9). Taking a deeper look into our patients, the CT scans of their chests showed typical findings of SAVI; however, the sequence of their symptoms does not align with the natural course of the disease, as described in the majority of cases reported. The patients reported here seemed to have acquired lung involvement far before the other clinical features of SAVI. We assume that this atypical presentation of a relatively rare genetic disease is the reason behind the delayed genetic testing and diagnosis.

When comparing the two siblings with the other reported cases globally, we hypothesize that the novel homozygous nature of their mutation is what strikingly resulted in a milder form of disease; this opposed the expected genetic outcome based on the standard concepts of homozygous vs heterozygous genetic mutations. Of the reported cases, all had heterozygous mutations, some of which later developed nasal septal perforation, had a digit amputated or even died with pulmonary complications (2, 9). However, these siblings, despite carrying two copies of the mutation on each gene, have not exhibited such a progression in their diseases thus far. Building on all the cases of SAVI reported in the literature, we now are closer to understanding the pathophysiology that leads to such a syndrome.

Ongoing research in recent years has led to a significant advancement in understanding the function of the *STING1* gene and its effect on *STING*. This understanding plays a key role in developing newer treatment options that are more effective in targeting the specific underlying immunological overactivation (6). The traditional therapeutic approach with multiple immune-suppressive medications concurrently as well as a single drug have both failed to elicit significant clinical improvement or inhibition in disease progression (2, 6, 7). Janus kinases (JAK), a family of enzymes, play a key role in the signaling cascade on cytokine receptors type I and II. These cytokines control immune responses and cellular proliferation once activated (23). Another sequalae of cytokine activation *via* JAK is the phosphorylation of signal transducers and activators of transcription 1 and 3 (STAT1 and STAT3), a family of DNA binding proteins, which further translocate into the nucleus and activate gene regulation and interferon production (21, 24). Such an understanding has promoted the development of JAK inhibitors (JAK-Is), a group of medications designed to manage inflammatory, autoimmune diseases and neoplasms caused by overactivation of the STING pathway. JAK-Is are designed to target JAK 1, 2, and 3 and TYK2 depending on their mechanism of action and clinical significance (21). **Supplementary Table 3** summarizes the mechanisms and uses for JAK-Is.

With greater insight into the disease background of SAVI and the indirect role of JAK-Is on the interferon signaling pathway, a newer approach utilizing JAK-Is has been tried. Such medication, despite having no direct effect on STING, has led to a well-desired reduction in severity alongside halting progression of disease through the inhibition of its inflammatory nature by suppressing the gene encoding for interferon-β (*IFNβ1*).

To the best of our knowledge, from all reported cases of SAVI, to date, only eight have utilized one of the JAK-Is as therapy, as shown in **Supplementary Table 2** Comparing the effectiveness of JAK-Is on patients with SAVI, we believe that the clinical and immunological improvements outlined in disease progression correlate with objective improvement in measured cardiac function and pulmonary artery pressure despite permanent radiological changes secondary to fibrosis exhibited in both lung CT scans and ECHO reports. Our experience with the use of JAK-Is in both siblings is similar to the outcome reported with other treated patients with SAVI. However, we could not compare the change in INF-beta levels seen in pre- and posttreatment settings in our patients and the others treated with a JAK-I due to lack of standardized reporting technique. Although in this paper the functional effect of the R281W variant was not tested, the effect of the same mutation was recently confirmed by Lin et al. (22).

The discovery of this novel homozygous mutation may contribute to a better understanding of STING regulatory mechanisms, and, more importantly, may help developing therapeutic approaches targeting this pathway.

## Data Availability Statement

The original contributions presented in the study are included in the article/**Supplementary Material**. Further inquiries can be directed to the corresponding author.

## Ethics Statement

This study was approved by the ethics committee at King Saud University (KSU) Institutional Review Board (Reference number 19/266/IRB). Written informed consent to participate in this study was provided by the participants’ legal guardian/next of kin. Written informed consent was obtained from the individual(s) and minor(s)’ legal guardian/next of kin, for the publication of any potentially identifiable images or data included in this article.

## Author Contributions

MA, JM, and RH designed the research. MA, JM, MA-A, and NA performed the research. MA, JM, NA, TK, RH, FJ-V, NSS-A, and SA analyzed and interpreted the data and wrote the paper. MA, TK, NSS-A, and RH critically revised the article. All authors commented on the manuscript. All authors contributed to the article and approved the submitted version.

## Conflict of Interest

The authors declare that the research was conducted in the absence of any commercial or financial relationships that could be construed as a potential conflict of interest.

## Abbreviations

SAVI, STING-associated vasculopathy of infantile-onset (SAVI); JAK-I, Janus kinase inhibitor.
