# Supplementary material for: A Novel Biallelic STING1 Gene Variant Causing SAVI in Two Siblings
Source: Front Immunol. 2021 Jan 8;11:599564. doi: 10.3389/fimmu.2020.599564 (PMC7820697; doi:10.3389/fimmu.2020.599564)
Supplement: Supplementary file 1 [file Table_1.doc]

**Supplementary Table 1.** List of primers used in this study.

| **Gene name** | **Primer sequence** |
| --- | --- |
| NG_034249.1 Homo sapiens transmembrane protein 173 (TMEM173) | Forward primer- ACCTCCCAGGACTCTATCGT |
|  | Reverse primer-CCTGCCCTCCAGCCTATCAA |
| NM_024013.2 Homo sapiens interferon alpha 1 (IFNA1) | Forward primer-AGGAGGAAGGAATAACATCTGGT |
|  | Reverse primer-AGCAGGGGTGAGAGTCTTTG |
| NM_002176.3 Homo sapiens interferon beta 1 (IFNB1) | Forward primer-GCAGTTCCAGAAGGAGGACG |
|  | Reverse primer-TAGTCTCATTCCAGCCAGTGC |
| NM_001243211.1 Homo sapiens interleukin 18 (IL18) | Forward primer-AAACCTGGAATCAGATTACTTTGG |
|  | Reverse primer-GTCCGGGGTGCATTATCTCT |

**Supplementary Table 2.** Reported cases with SAVI treated with JAK-I with reported clinical outcome

| Study | Manifestations | | | | | | Rx | Outcome | | | | | Genetics | |
| --- | --- | --- | --- | --- | --- | --- | --- | --- | --- | --- | --- | --- | --- | --- |
|  | Lung (ILD) | P  HTN | Skin/acral necrosis | Joints | Vasculitis | Inflammatory markers | JAK-I | Skin | Lungs | Growth | Biochemical | Death | Allele status | Genotype |
| 1. Chia et al.(1) | (+) | NR | (+) | (+) | NR | ESR: 89  IgE: 283  IgG: 4747 mg/dL | Baricitinib | NR | NR | NR | NR | Alive | Heterozygous | c.461A>G p.N154S |
| 2. Seo et al.(2) | (+) | NR | (+) | NR | (+) | NR | Tofacitinib 5 mg OD | Improved | Static | NR | NR | Alive | Heterozygous | c.304T>C p.Ser102Pro  c.835T>C p.Phe279Leu |
| 3. Yu et al.(3) | (+) | (+) | (+) | NR | NR | CRP: 21  ESR: 78  IgG: 22.16 g/L | Tofacitinib 5 mg BD | NR | NR | NR | NR | NR | Heterozygous | c.463G>A V155M |
| 4. Liu et al. ^(4)^ | (+) (5/6) | NR | (+) (6/6) | (+) (1/6) | (+) (6/6) | NR | Tofacitinib, ruxolitinib, and baricitinib (in vitro) | NR | NR | NR | NR | Alive (4/6) | Heterozygous | p.N154S, p.V155M, p.V147L  p. V155R |
| 5. Saldanha et al. ^(5)^ | (+) | (+) | (+) | NR | (-) | CRP: 25  ESR: 34  Ferritin: 360 µg/L  IgA: 1.09 g/L  IgM: 2.39 g/L  IgG: 11.1 g/L | Ruxolitinib 5 mg OD | NR | NR | Improved | CRP: <1  ESR: 11  Ferritin: NA  IgA: 1.12 g/L  IgM: 0.62 g/L  IgG: 10.10 g/L | Alive | Heterozygous | c.852G>T R284G |
| 6. Sanchez et al.(6) | (4/4) (+) | NR | (3/4) (+) | NR | (4/4) (+) | NR | Baricitinib | Improved flares and halted acronecrosis | Static with no further decline in PFT readings | Improved (bone age) | NR | NR | Heterozygous | NR |
| 7. Balci et al.(7) | (+) | NR | (+) | NR | (+) | ESR: 69  CRP: 7.9 | Baricitinib 2 mg daily | Improved | Improved | NR | ESR: 50  CRP: 1.5 | Alive | Heterozygous | c.461A>G  N154S |
| 8. Volpi et al. (8) | P1: (+) | (+) | (+) | (+) |  | CRP: 0-4.2  ESR: 48-57  IGA: 274 mg/dl  IgG: 1631 mg/dl  IgM: 195 mg/dl | Ruxolitinib | Improved | NR | Static | CRP: 0  ESR: 30 | Alive | Heterozygous | c.463G>A p.Val155 Met |
|  | P2: (+) | (+) | (+) | (-) | (-) | CRP: 0-15  ESR: 23-24  IGA: 340 mg/dl  IgG: 1639 mg/dl  IgM: 1639 |  | Lesions were transient from the start | Improved SPO_2_, walking distance and FVC. Worse chest CT | Improved from 3^rd^-5^th^ centile to 10^th^ to 25^th^ centile (wt) | CRP:0  ESR: 23 | Alive |  | c.842G>A p.Arg281Gln |
|  | P3: (+) | NR | (+) | (-) | NR | CRP: 12-34 ESR: 20-79  IGA: 130 mg/dl  IgG: 1674 mg/dl  IgM: 266 mg/dl |  | Lesions were transient from the start | NR | Improved from 3^rd^-10^th^ centile to 25^th^ centile (wt) | CRP:2.28  ESR: 10.6 | Alive |  | c.461A>G p.Asn154Ser |

Key: (NR) not reported

(+) Yes/(-) No

P: patient

ESR: erythrocyte sedimentation rate (mm/h)

CRP: C-reactive protein (mg/dL)

***Supplementary Table 3.***  *JAK-Is and Their JAK Isoform(s) Inhibited, Uses and Current Drug Status.*

| Medication | JAK isoform(s) Inhibited | Use | Drug status |
| --- | --- | --- | --- |
| Ruxolitinib | JAK1, JAK2 | Psoriasis, MF, RA | FDA approved |
| Tofacitinib | JAK 1, JAK3 | RA. Psoriasis | FDA approved |
| Baricitinib | JAK1, JAK2 | RA | Approved |
| Upadacitinib | JAK1 | RA | FDA approved |
| Filgotinib | JAK1 | RA, CD | Ongoing clinical trial |
| Peficitinib | JAK1, JAK2, JAK3 | RA | Approved in Japan |
| Decernotinib | JAK3 |  | Clinical trial phase II |
| Oclacitinib | JAK1, JAK2 | RA |  |
| Abbreviations: RA: Rheumatoid arthritis, MF: Myelofibrosis, CD: Crohn’s disease | | | |

**References**

1. Chia J, Eroglu FK, Ozen S, Orhan D, Montealegre-Sanchez G, de Jesus AA, et al. Failure to thrive, interstitial lung disease, and progressive digital necrosis with onset in infancy. J Am Acad Dermatol. 2016;74(1):186-9.

2. Seo J, Kang JA, Suh DI, Park EB, Lee CR, Choi SA, et al. Tofacitinib relieves symptoms of stimulator of interferon genes (STING)-associated vasculopathy with onset in infancy caused by 2 de novo variants in TMEM173. J Allergy Clin Immunol. 2017;139(4):1396-9.e12.

3. Yu ZX, Zhong LQ, Song HM, Wang CY, Wang W, Li J, et al. [Stimulator of interferon genes-associated vasculopathy with onset in infancy: first case report in China]. Zhonghua Er Ke Za Zhi. 2018;56(3):179-85.

4. Liu Y, Jesus AA, Marrero B, Yang D, Ramsey SE, Montealegre Sanchez GA, et al. Activated STING in a Vascular and Pulmonary Syndrome. New England Journal of Medicine. 2014;371(6):507-18.

5. Saldanha RG, Balka KR, Davidson S, Wainstein BK, Wong M, Macintosh R, et al. A Mutation Outside the Dimerization Domain Causing Atypical STING-Associated Vasculopathy With Onset in Infancy. Front Immunol. 2018;9:1535.

6. Sanchez GAM, Reinhardt A, Ramsey S, Wittkowski H, Hashkes PJ, Berkun Y, et al. JAK1/2 inhibition with baricitinib in the treatment of autoinflammatory interferonopathies. J Clin Invest. 2018;128(7):3041-52.

7. Balci S, Ekinci RMK, de Jesus AA, Goldbach-Mansky R, Yilmaz M. Baricitinib experience on STING-associated vasculopathy with onset in infancy: A representative case from Turkey. Clin Immunol. 2020;212:108273.

8. Volpi S, Insalaco A, Caorsi R, Santori E, Messia V, Sacco O, et al. Efficacy and Adverse Events During Janus Kinase Inhibitor Treatment of SAVI Syndrome. J Clin Immunol. 2019;39(5):476-85.
